# Does persistent active trachoma mandate antibiotic mass drug administration? A comparison of prevalence of trachomatous inflammation–follicular with that of conjunctival infection and anti-chlamydial antibodies, Western Province, Zambia

**DOI:** 10.1093/inthealth/ihaf092

**Published:** 2025-08-26

**Authors:** Consity Mwale, Chileshe Mboni, Ngonda Saasa, Chummy S Sikasunge, Chisanga Chelu, Phyllis M Moonga, Grace Hameja, Levison Nkhoma, Frank Shamilimo, Namasiku S Kunda, Glenda Mulenga, Tabonga Naluonde, Nicholas Mutale, Sarah Boyd, Rosemary Pearson-Clarke, Ana Bakhtiari, Cristina Jimenez, Emma M Harding-Esch, Michael Dejene, Katherine Gass, Katrina Farber, Mohamed Bah, Caleb Mpyet, Freddie Masaninga, Nathan Nsubuga Bakyaita, Mwila Lundamo, Grace Chipalo Mutati, Sikufele Mubita, Davison J Kwendakwema, Paul Courtright, Anthony W Solomon, Kangwa I M Muma

**Affiliations:** Kitwe Teaching Eye Hospital, Administration Department, P.O. Box 20969, Kitwe 10101, Zambia; Lusaka Apex Medical University, Faculty of Medicine, P.O. Box 31909, Lusaka 10101, Zambia; Kitwe Teaching Eye Hospital, Administration Department, P.O. Box 20969, Kitwe 10101, Zambia; Departments of Disease Control and Paraclinical Studies, School of Veterinary Medicine, University of Zambia, P.O. Box 32379, Lusaka 10101, Zambia; Departments of Disease Control and Paraclinical Studies, School of Veterinary Medicine, University of Zambia, P.O. Box 32379, Lusaka 10101, Zambia; Kabwe Central Hospital, Eye Unit, P.O Box 80917, Kabwe 10101, Zambia; Ministry of Health, Directorates of Public Health and Clinical Care, P.O. Box 30205, Lusaka 10101, Zambia; Ministry of Health, Directorates of Public Health and Clinical Care, P.O. Box 30205, Lusaka 10101, Zambia; Ministry of Health, Directorates of Public Health and Clinical Care, P.O. Box 30205, Lusaka 10101, Zambia; Ministry of Health, Directorates of Public Health and Clinical Care, P.O. Box 30205, Lusaka 10101, Zambia; Ministry of Health, Directorates of Public Health and Clinical Care, P.O. Box 30205, Lusaka 10101, Zambia; Sightsavers, P.O. Box 37535, Lusaka 10101, Zambia; Lions Aid, P.O. Box 37166, Lusaka 10101, Zambia; Lions Aid, P.O. Box 37166, Lusaka 10101, Zambia; International Trachoma Initiative, The Task Force for Global Health, 330 West Ponce de Leon Ave., Decatur, GA 30030, USA; International Trachoma Initiative, The Task Force for Global Health, 330 West Ponce de Leon Ave., Decatur, GA 30030, USA; International Trachoma Initiative, The Task Force for Global Health, 330 West Ponce de Leon Ave., Decatur, GA 30030, USA; Neglected Tropical Diseases Department, Sightsavers, Haywards Heath RH16 3BW, UK; Clinical Research Department, London School of Hygiene and Tropical Medicine, London WC1E 7HT, UK; Neglected Tropical Diseases Department, Sightsavers, Addis Ababa 495, 1110, Ethiopia; Neglected Tropical Diseases Support Centre, Task Force for Global Health, Decatur, GA 30030, USA; Neglected Tropical Diseases Support Centre, Task Force for Global Health, Decatur, GA 30030, USA; Neglected Tropical Diseases Support Centre, Task Force for Global Health, Decatur, GA 30030, USA; Sightsavers, Nigeria Country Office, Kaduna 930222, Nigeria; Department of Ophthalmology, University of Jos, Jos 930222, Nigeria; World Health Organization, Lusaka 10101, Zambia; World Health Organization, Lusaka 10101, Zambia; Lusaka Provincial Health Office, P.O. Box 32573, Lusaka 10101, Zambia; Mainasoko Medical Centre Eye Department, P.O. Box 320091, Lusaka 10101, Zambia; Zambia Statistics Agency, Population and Demography Division, P.O. Box 31908, Lusaka 10101, Zambia; Beverly Eye and Dental Clinic, Ndola, Zambia; KCCO, Division of Ophthalmology, University of Cape Town, Cape Town 7925, South Africa; Global Neglected Tropical Diseases Programme, World Health Organization, Geneva 1211, Switzerland; Phil Eye Hospital, 30 Pamo Avenue, Kitwe 10101, Zambia; School of Medicine, University of Zambia, P.O. Box 50110, Lusaka 10101, Zambia; School of Medicine, Levy Mwanawasa Medical University, P.O. Box, 33991, Lusaka 10101, Zambia

**Keywords:** *Chlamydia trachomatis*, persistent active trachoma, safe strategy, serological and PCR markers, trachomatous trichiasis

## Abstract

**Background:**

The evaluation unit comprising Kaoma, Luampa, and Nkeyema districts, Western Province, Zambia, has persistent active trachoma. In 2023, we sought to compare the evaluation unit–level prevalence of the active trachoma sign, trachomatous inflammation–follicular (TF), to that of conjunctival *Chlamydia trachomatis* (Ct) infection and anti-*Chlamydia trachomatis* (Ct) seropositivity.

**Methods:**

We conducted a cluster-sampled cross-sectional survey. In selected households, we examined all consenting residents ≥1 y of age for trachoma. We collected dried blood spots (DBSs) by finger-prick from children ages 1–9-y and conjunctival swabs from the left eyes of children ages 1–5-y. DBSs were tested for antibodies to the *C. trachomatis* antigen Pgp3 by lateral flow assay. We tested conjunctival swabs for *C. trachomatis* DNA by GeneXpert polymerase chain reaction (PCR).

**Results:**

The TF prevalence in children ages 1–9-y was 9.2%. In children ages 1–5-y, anti-Pgp3 seroprevalence was 1.7% and the seroconversion rate was 0.6 per 100 person-years. The prevalence of conjunctival *C. trachomatis* DNA in children ages 1–5-y was 0%.

**Conclusions:**

Based on TF prevalence, this population qualified for additional antibiotic mass drug administration rounds, but PCR and serology—more specific indicators of current or recent *C. trachomatis* infection than TF—confirmed an absence of significant current community *C. trachomatis* transmission, allowing a transition to surveillance. Adding these indicators is helpful in persistent active trachoma.

## Introduction

Trachoma is a disease caused by recurrent, chronic *Chlamydia trachomatis* infection of the conjunctiva. A neglected tropical disease, it is the leading infectious cause of blindness, primarily affecting marginalised communities that have poor access to water, sanitation, and healthcare. Repeated infections lead to scarring of the inner surface of the eyelid, causing eyelashes to turn inward and scratch the eyeball (trachomatous trichiasis [TT]), which can damage the cornea and ultimately result in blindness. There is a global initiative to eliminate trachoma as a public health problem by 2030 using the World Health Organization (WHO)-recommended SAFE strategy: Surgery to address TT and prevent blindness, Antibiotics to clear infection, Facial cleanliness and Environmental improvement—particularly increased access to clean water and appropriate sanitation—to reduce transmission.^1,2^ Programmatic targets include a reduction in the prevalence of trachomatous inflammation–follicular (TF) in children ages 1–9-y (TF_1–9_) to <5% and of TT in individuals ≥15-y of age to <0.2%, within previously endemic evaluation units (EUs), which are usually administrative districts of 100 000–250 000 persons.^3^

Reduction in TF_1–9_ is primarily accomplished through annual rounds of antibiotic mass drug administration (MDA), the ‘A’ in SAFE, and implementation of F and E. Trachoma impact surveys (TISs) are conducted to assess TF_1–9_ after the planned number of rounds of MDA.^4^ If TF_1–9_ is <5%, then a trachoma surveillance survey (TSS) is conducted at least 2 years later to ensure TF_1–9_ remains at <5%.^5^ However, a number of EUs worldwide have not achieved or maintained TF_1–9_ at <5%, despite numerous annual rounds of MDA. At a 2021 WHO informal consultation to address this issue, the following definitions were proposed: an EU with persistent active trachoma is one in which at least two TISs estimate TF_1–9_ as ≥5% and in which TF_1–9_ <5% has never been reported; and an EU with recrudescent active trachoma is characterised by at least one TSS with TF_1–9_ ≥5%.^6^ It was recommended to conduct enhanced surveys in such EUs, including estimating complementary indicators of *C. trachomatis* infection, such as antibody and nucleic acid amplification tests on conjunctival swabs.^6^ These complementary indicators could help identify TF due to causes other than ocular *C. trachomatis* infection or systematic grading errors, either of which might inflate TF prevalence and unnecessarily prolong MDA.

Zambia, like other sub-Saharan African countries, has faced significant challenges with trachoma, particularly in rural and impoverished areas.^7–13^ Through coordinated efforts by the Zambian Ministry of Health, other government ministries, international organisations, and local stakeholders, Zambia is making excellent progress in reducing the number of people who need interventions against trachoma. MDA campaigns (using azithromycin donated by Pfizer through the International Trachoma Initiative^14^) have played a critical role, along with community-driven hygiene and sanitation initiatives. Despite this progress, persistent active trachoma is an emerging challenge in some populations, including the EU composed of Kaoma, Luampa, and Nkeyema districts of the Western Province.

The baseline TF_1–9_ in this EU, estimated in 2008, was 32.7%. The TISs undertaken in 2017 and 2018 returned TF_1–9_ estimates of 10.7% (95% confidence interval [CI] 7.4 to 14.3) and 11.3% (95% CI 7.2 to 16.0), respectively (Supplementary Table 1). In between and after these time points, spanning the period 2012–2022, a total of nine rounds of MDA in this EU were implemented, with antibiotic coverage estimates exceeding the WHO-recommended minimum of 80% of the population^15^ on each occasion (Supplementary Table 2). The EU was therefore classified as having persistent active trachoma.^6^

This EU could benefit from modified approaches in order to speed up the national goal of trachoma elimination.^6^ In particular, the Ministry of Health identified the need for better surveillance. Here, we report data from a 2023 enhanced TIS (‘TIS-Plus’) that included serology and testing for infection in these three districts.

## Methods

### Study design

We employed a three-stage cluster sampling methodology, encompassing the sampling of wards within districts, clusters within wards, and homes within clusters. Wards, which have well-defined geopolitical boundaries, population sizes and structures, are the most basic administrative unit for which complete lists were centrally available. The estimated populations of Kaoma, Luampa, and Nkeyema in 2022^16^ (when survey planning was being undertaken) are shown in Table 1.

**Table 1.** Population data and number of subdivisions of three districts (surveyed as one evaluation unit)^16^

Province	District	Population	Population of 1- to 9-y-olds	Number of wards
Western	Kaoma	146 690	38 724	18
	Luampa	61 023	19 746	12
	Nkeyema	106 074	30 254	8

### Sample size calculation

The single population proportion for precision formula was used to estimate the required sample size. We calculated this as follows: n=DEFF×(z^2^×p(1−p)/c^2^)×e, where DEFF (2.63) is the design effect,^17^ z (1.96) is the standard deviation for 95% CIs, p (4%) is the expected prevalence, c (±2%) is the absolute precision and e (1.2) is an inflation factor to compensate for non-response.^18^ Consequently, we sought to enumerate a total of 1164 children aged 1–9-y.

### Determination of the number of clusters in the evaluation unit

To calculate the necessary number of clusters for the survey, the targeted number of children aged 1–9-y was divided by the mean number of households efficiently visited by one team each day (n=30) multiplied by the average number of children aged 1–9-y per household. The mean number of children aged 1–9-y in Zambian households is 1.63. Thus, a total of 24 clusters were expected to be required (1164/[30×1.63]) (Table 1).

### Sampling of wards, clusters, and households

First, wards were selected using probability proportional to size (PPS) through systematic sampling. Second, 24 clusters were selected using PPS within designated wards. Third, households within each selected cluster were selected by compact segment sampling. A lottery sampling procedure was used for the latter process, identifying the final cohort of 30 households in each cluster.

### Training of survey teams

The training of graders and recorders occurred over five days using the most recent Tropical Data training system, as outlined in the Tropical Data training manuals.^19^ Additional training was conducted for sample collection/management for dried blood spots (DBSs) and swabs. Each survey team comprised a grader, recorder, laboratory technician, tuber, driver, and local guide.

For the 2023 survey, graders were assessed based on three classroom assessments: (i) a follicle identification test; (ii) photo-based intergrader agreement (IGA) tests; and (iii) classroom objective structured clinical examinations (OSCE) to evaluate examination procedures. Subsequently, they executed a field-based OSCE.

Recorders were instructed to precisely document household and individual examination data on Android smartphones and had to successfully complete a recorder assessment exercise. Competent graders and recorders subsequently trained together before joining the survey team. Two ophthalmologists supervised the teams in the field, dedicating at least one day/week to offer practical technical assistance to each survey team.

### Field management and ownership

All data were recorded electronically in the Tropical Data app, a customised version of Open Data Kit (ODK) Collect, accessible at no cost on the Google Play Store (https://play.google.com/store/apps/details?id=com.tropicaldata.collect.android). Acquired data were retained on the smartphone's internal memory until internet connectivity was established, after which it was uploaded to a Cloud-based server accessible 24/7 exclusively by designated personnel of the Ministry of Health. Data were available through the password-protected Tropical Data website (https://www.tropicaldata.org/projects). The Ministry of Health authorised, validated, and approved data collection and quality.

### Data collection

Household Global Positioning System (GPS) coordinates were collected. This was followed by collection of data on water, sanitation, and hygiene (WASH) through structured interviews with the household head or their representative. Visual assessment of domestic WASH facilities, including latrines and water sources where applicable, was also conducted.

All people ≥1-y of age residing in sampled households were eligible for inclusion. The grader looked for TT and indicators of active trachoma (TF and/or trachomatous inflammation–intense [TI]). In individuals with TT, examination was also undertaken for trachomatous scarring (TS). Trichiasis was documented separately for the upper and lower eyelids and the grader counted the number of eyelashes in contact with the eyeball and the quantity of recently epilated eyelashes from the affected eye, relaying the counts to the recorder. The examiner used 2.5× magnifying binocular loupes, together with sunlight or a torch for illumination. Follicle size guides were employed to assist in the diagnosis of TF.^20^

Individuals with trichiasis were queried further to assess their access to information and services regarding TT surgery in order to ascertain whether TT had already been recognised by the health system. For TT management questions, a health worker was defined as anyone who was able to offer access to primary healthcare services.

Individuals diagnosed with active trachoma or any other presumed bacterial ocular condition were given two tubes of 1% tetracycline eye ointment at no cost. Patients with TT or other ocular problems were referred for further management.

### Specimen collection and storage

#### Conjunctival swab collection

Left conjunctiva swab samples were collected from all examined children aged 1–5-y. (The left eye was chosen arbitrarily; we had no reason to believe that systematic differences in infection status would exist between the right and left eyes.) Barcodes were used to link the ocular swabs with the clinical examination data and the DBS samples. For every 20th child, a negative field control swab (‘air swab’) was collected. In this case, a sterile swab was passed within an inch of the child's conjunctiva without touching the child.

The swabs were stored in microcentrifuge tubes, and placed in the sample collection box, located in the cooler bag filled with frozen ice packs. Swab samples were transported on ice in a closed, insulated container (approximately 4°C) immediately after completion of field work until arrival at the district hospital laboratory for storage at 4°C for 3 days before they were sent to the University of Zambia School of Veterinary Medicine (UNZA-Vet) laboratory for storage at −20°C until testing was conducted.

#### Dry blood spot collection

Finger prick blood was collected onto filter paper containing six extensions, each calibrated to absorb 10 µl of blood (TropBio Pty Ltd, Townsville, Queenland, Australia). Filter papers were dried overnight, placed in individual plastic bags, and then stored together in a bag with desiccant, after which they were stored at 4°C for three days. The resulting DBSs were transported to UNZA-Vet where they were stored at −20°C until testing.

### Laboratory testing

#### Pgp3 lateral flow assay (LFA)

DBSs were tested by lateral flow assay (LFA) using methods described previously.^21^ Briefly, Pgp3 black latex conjugate (Abcam, Cambridge, United Kingdom) and streptavidin gold conjugate (Arista Biologicals, Allentown, PA, USA) were diluted 1:120 in phosphate-buffered saline with Tween-20 (PBST 0.3% Tween-20 in 1× PBS) to create a conjugate master mix. DBSs were eluted in 60 µl of conjugate master mix and incubated overnight at 4°C. The next day, LFA dipsticks were added to each well and incubated for 15 min. PBST (80 µl) was then added to each well for approximately 5 min. LFA dipsticks were removed and read as positive, negative or invalid. External controls (two positive; one negative) were run once per week.

#### Cepheid GeneXpert

Ocular swabs were tested using Cepheid GeneXpert CT/NG kits (Cepheid, Sunnyvale, CA, USA) according to previously published methods.^22^ Each swab was eluted in 1 ml of diethyl pyrocarbonate water and left to stand for 1 h at 2–8°C. Each swab elution (200 µl) was transferred to a tube containing Cepheid transport media. The swabs were grouped in pools of five samples according to clusters. Where the number of swabs in the cluster were not divisible by five, swabs from other clusters were added to the pool(s) to form a complete pool of five samples. The pooling was done by staff who were blinded to the testing process. Pools of five specimens (260 µL each elution/transport media mixture) were created in a 2 mL tube for a total volume of 1200 µl. This pool was immediately added to the sample chamber of the Cepheid CT/NG cartridge and run according to the manufacturer's instructions. The samples from any positive pools were rerun individually to identify the individual(s) that was positive. Air swabs were separated from the rest of the swabs and tested in their own in pools of five according to cluster. The air swabs were eluted, pooled and tested following the same procedure as the ocular swabs. Each run included multiple kit controls: an internal sample processing control, sample adequacy control and probe check control. External controls were tested once per week.

### Statistical analysis

Clinical data were analysed in R using standard Tropical Data methodologies.^18,23^ In summary, the EU-level TF_1–9_ prevalence was determined by calculating the mean of cluster-level proportions adjusted for age in one year intervals (based on the latest census data). The prevalence of TT in individuals ≥15-y of age was similarly computed, but the cluster-level proportions were adjusted for gender and age in five-year intervals. The WASH data were evaluated according to the classifications established by the WHO/UNICEF Joint Monitoring Program.^24^ The Ministry of Health approved the final data analysis report.

EU-level seroprevalence estimates were calculated using a generalized linear model. Seroconversion rates (SCRs) per 100 person-years were estimated using a generalized linear model with a complementary log–link and robust standard errors.^25^  *C. trachomatis* infection prevalence was calculated by determining the number of polymerase chain reaction (PCR)-positive swabs, based on the results of the individual assays run from the PCR-positive pools, divided by the total number of ocular swabs tested.

## Results

Fieldwork took place from September to October 2023. After completing work in 24 clusters, 902 consenting 1- to 9-year-olds had been enrolled and examined. Two additional clusters were therefore added to the 24 originally selected, in order to meet our sample size requirement.

**Figure 1.**
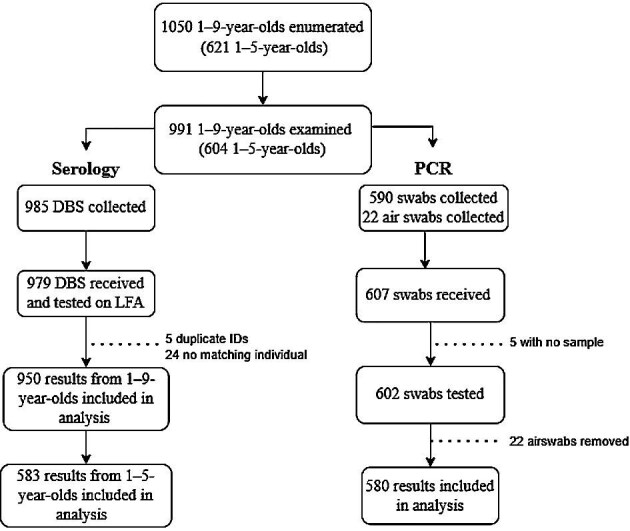
Flow chart of samples collected and tested by each assay. ‘Five with no sample’ means that sample identification numbers appeared on the manifest but no samples were available.

Across 26 clusters, a total of 1050 1- to 9-year-olds were enumerated in selected households. Of these individuals, 991 were examined and 985 had DBSs collected. A total of 590 1- to 5-year-olds had conjunctival swabs collected (Figure 1). The numbers of samples tested by LFA and PCR are also shown in Figure 1. TF_1–9_ was 9.2% (95% CI 6.0 to 13.1). The seroprevalence was 5.4% (95% CI 4.1 to 7.0) in 1- to 9-year-olds and 1.7% (95% CI 0.9 to 3.2) in 1- to 5-year-olds. The seroconversion rate (SCR) per 100-person years was 1.2 (95% CI 1.1 to 1.3) in 1- to 9-year-olds and 0.6 (95% CI 0.4 to 0.8) in 1- to 5-year-olds. All conjunctival swabs tested negative for *C. trachomatis* DNA.

The prevalence of TT unknown to the health system in ≥15-year-olds was 0.4% (95% CI 0.1 to 0.9).

Some 55% of households had access to an improved source of water for drinking. Nearly half of all households had an improved drinking water source where collection time was <30 minutes for a round trip, including queuing time. Including both improved and unimproved drinking water sources, 87% of households had a drinking water source where collection time was <30 minutes for a round trip, including queuing time. Less than 1% of households had access to an improved latrine: 42% of households reported usually disposing of adult human faeces outside somewhere; 37% usually used a shared or public latrine or toilet; and 20% usually used a private latrine or toilet.

## Discussion

Significant progress has been made against trachoma worldwide. By April 2025, 21 countries had been validated by the WHO as having eliminated trachoma as a public health problem.^26^ Zambia has not lagged behind this progress elsewhere, having reduced the number of districts nationally in which trachoma is a public health problem from 56 at baseline to 15 in 2023, but some challenges remain. The first baseline surveys were conducted in 2007,^9^ yet work to eliminate trachoma in Zambia is still needed. To further accelerate elimination efforts, the national trachoma elimination programme has proactively enhanced its implementation of the SAFE strategy. Several areas now require innovative approaches using integrated multi-sectoral primary healthcare interventions,^27^ potentially including documented full geographical coverage for TT case-finding,^28^ expanded WASH access, more-frequent-than-annual MDA,^6,29,30^ the use of TIS-Plus,^29,31^ and knowledge, attitudes, and practices research. The work described in this manuscript is an example of such innovation.

Our results allow some programmatically important conclusions to be drawn. The serological data, indicative of exposure at any time in an individual's life, were consistent with very low intensity or absent conjunctival *C. trachomatis* transmission: the SCR (0.6 in 1- to 5-year-olds) fell well below the proposed threshold for intervention that emerged from a recent multi-national analysis of 48 similar serosurveys.^32^ PCR of conjunctival swab eluates (prevalence of PCR positivity 0%) confirmed the absence of any current community *C. trachomatis* transmission. These insights into transmission would have evaded us if we had not taken swabs and DBSs, leaving us looking at a persistently high prevalence of the conjunctival sign TF (9.2%) as the sole guide to programmatic decision-making for the A, F and E components of SAFE. WHO currently recommends that where TF_1–9_ is ≥5%, as it was in this EU, that antibiotic MDA for trachoma elimination purposes should be continued,^15^ but in the face of evidence of absence of significant population-level conjunctival *C. trachomatis* transmission, it seems unnecessary to continue to periodically offer the whole population anti-*C. trachomatis* antibiotics.^33^ The TT data indicate that conjunctival *C. trachomatis* infection previously contributed to complications of trachoma in the area, providing reassurance that prior rounds of MDA were warranted. However, continuing antibiotics in this EU is now difficult to justify.

The results of this study have led to a change in strategy at the Zambian Ministry of Health. In this EU, further MDA is now being withheld while implementing the other components of the SAFE strategy. A TSS-Plus will be conducted at least two years after the TIS-Plus described here.

There were several strengths to this study. We used a standardised survey approach in accordance with WHO recommendations^34^ and included a raft of quality assurance and quality control mechanisms that are routinely implemented for trachoma prevalence estimation.^18,20,23,35,36^ Our graders were Tropical Data–certified and highly experienced, instilling confidence in the accuracy of the examination data. And our serological work was co-designed and overseen by the assay developers at the US Centers for Disease Control and Prevention.^37–42^ A limitation of our survey design was our restriction of conjunctival swab collection to 1- to 5-year-olds. Prevalence and load of conjunctival *C. trachomatis* is highest in pre-school age children,^43^ so this age range allowed us to maximize the chance of identifying current infection if present, whilst containing costs. The broader age range in which we estimated TF and antibody prevalence prevents direct comparison between the three indices, but the primary purpose of this work was to help guide future programmatic action. We hope it will also be able to inform work elsewhere in Zambia and beyond.

Persistent trachoma could conceivably be driven by one or more factors, varying by context, including inadequate access to clean water and sanitation,^44^ undermining efforts to maintain proper facial hygiene and environmental cleanliness. Poor living conditions and close contact with infected individuals might contribute to ongoing community reinfection, while gaps in antibiotic MDA coverage could leave certain parts of the population untreated, allowing infection to persist and spread again. Additionally, cultural and behavioural factors, such as inadequate education and resources for latrine construction, can contribute to persistent active trachoma. Eliminating persistent active trachoma requires tailored approaches that address these underlying factors.^6^ Our data on access to WASH, particularly sanitation, suggest that the population in the EU studied here is under-served, but ongoing *C. trachomatis* transmission secondary to inadequate WASH access is manifestly not the issue locally. Strengthening community engagement on WASH^45^ to facilitate expansion in access to clean water and sanitation, continuing antibiotic MDA^46,47^ and improving the integration of trachoma programme into broader health systems may be important in other EUs for Zambia to achieve and sustain the elimination target. For Kaoma, Nkeyema and Luampa, however, it seems that the active trachoma target itself should be adjusted, since *C. trachomatis* is not likely to be responsible for the ongoing high TF_1–9_ numbers. Similar scenarios are being experienced elsewhere^48–53^ and now must be the focus of international review.^1,54^

## Supplementary Material

ihaf092_Supplemental_File

## Data Availability

The data utilised for this study and report may be obtained from the corresponding author. The data are owned by the Zambia Ministry of Health.
