# Supplementary material for: Does persistent active trachoma mandate antibiotic mass drug administration? A comparison of prevalence of trachomatous inflammation–follicular with that of conjunctival infection and anti-chlamydial antibodies, Western Province, Zambia
Source: Int Health. 2025 Aug 26;18(2):274–81. doi: 10.1093/inthealth/ihaf092 (PMC13016739; doi:10.1093/inthealth/ihaf092)
Supplement: ihaf092_Supplemental_File [file ihaf092_supplemental_file.docx]

*Supplementary table 1: Outcomes of the previous trachoma impact surveys (TIS) in the evaluation unit composed of Kaoma, Luampa, and Nkeyema districts, Western Province, Zambia.*

| **Survey type (year)** | **Numbers of households visited, 1–9-year-olds examined and ≥15-year-olds examined** | **TF_1–9_ (95% CI)** | **TT_≥15_ (95% CI)** | **Households with an improved drinking water source (%)** | **Households with a drinking water source within a 30-min return journey of the household (%)** | **Household with an improved latrine (%)** |
| --- | --- | --- | --- | --- | --- | --- |
| TIS 1 (2017) | 722, 1038, 1317 | 10.7 (7.4–14.3) | 0.3 (0.0–0.6) | 42.7 | 37.4 | 0 |
| TIS 2 (2018) | 743, 1541, 1511 | 11.3 (7.2–16.0) | 0.3 (0.1–0.5) | 48.0 | 32.6 | 0.3 |

TF_1–9_ (prevalence of trachomatous inflammation—follicular, TF, in 1–9-year-olds): prevalence of TF adjusted for age in 1-year age bands; TT_≥15_ (prevalence of trachomatous trichiasis, TT, in ≥15-year-olds): prevalence of TT unknown to the health system adjusted for age and gender in 5-year age bands; in the surveys summarized in this table, TT included upper and/or lower eyelid trichiasis. CI=confidence interval, HH=household.

*Supplementary table 2: Estimated antibiotic mass drug administration coverage (number of individuals treated / estimated population from most recent census) for the evaluation unit composed of Kaoma, Luampa and Nkeyema districts, Western Province, Zambia, 2012–2022.*

| **Year** | **2012** | **2013** | **2014** | **2015** | **2016** | **2017** | **2019** | **2021** | **2022** |
| --- | --- | --- | --- | --- | --- | --- | --- | --- | --- |
| Coverage (%) | 94 | 80 | 88 | 85 | 89 | 101 | 85 | 114 | 108 |
